# The influence of neighborhood safety and built environment on childhood obesity: isolated and combined effect of contextual factors

**DOI:** 10.1590/0102-311XEN104822

**Published:** 2023-08-14

**Authors:** Ariene Silva do Carmo, Larissa Loures Mendes, Letícia de Oliveira Cardoso, Waleska Teixeira Caiaffa, Luana Caroline dos Santos

**Affiliations:** 1 Universidade Federal de Minas Gerais, Belo Horizonte, Brasil.; 2 Escola Nacional de Saúde Pública Sergio Arouca, Fundação Oswaldo Cruz, Rio de Janeiro, Brasil.

**Keywords:** Built Environment, Neighborhood Characteristics, Pediatric Obesity, Ambiente Construído, Características da Vizinhança, Obesidade Infantil, Entorno Construido, Características del Vecindario, Obesidad Infantil

## Abstract

This study aims to analyze the isolated and combined effect of objective measures concerning neighborhood safety, food, and physical activity environments on students’ obesity. This is a cross-sectional study conducted with 9- and 10-year-old children enrolled in the municipal education network of a Brazilian metropolis. Environment objective measures comprised neighborhood unsafety (annual criminality and road traffic accident rates), availability of public parks and spaces for physical activity practicing, and index of establishments that predominantly sell ultra-processed food. Euclidean buffers of 1,000m around the children’s house were used as eligible geographic units. This study adopted the Principal Component Analysis and Generalized Estimation Equation models. Stratified analyses were conducted based on neighborhood unsafety and on child’s family income. In total, 717 students were assessed, 12.2% of them were children with obesity. The latent variable of the obesogenic environment (deduced by environment unsafety rates and the index of establishments that predominantly sell ultra-processed food) was a risk factor for obesity in children with lower socioeconomic levels (OR = 2.37; 95%CI: 1.06-5.19). Public parks and spaces for physical activity practicing were protective factors against childhood obesity only in locations recording the lowest environment unsafety rates (OR = 0.30; 95%CI: 0.09-0.94). Based on our findings, social conditions change the effect of the environment on childhood obesity, reinforcing the relevance of inter-sectoral policies and strategies against this condition.

## Resumo

Este artigo busca analisar os efeitos isolado e combinado de medidas objetivas relativas à segurança da vizinhança, alimentação e ambientes de atividade física sobre a obesidade de crianças em idade escolar. Este estudo transversal foi realizado com crianças de 9 e 10 anos de idade que estavam matriculadas na rede municipal de ensino de uma metrópole brasileira. As medidas objetivas ambientais incluíram a insegurança nos bairros (índices anuais de criminalidade e acidentes de trânsito), disponibilidade de logradouros e espaços públicos para a prática de atividade física e o índice de estabelecimentos que comercializam majoritariamente alimentos ultraprocessados. Um *buffer* euclidiano de 1.000m em torno da casa das crianças foi tomado como unidade geográfica elegível. Nossa análise englobou os modelos de Análise de Componentes Principais e de Estimação de Equação Generalizada. Análises estratificadas foram realizadas com base na insegurança da vizinhança e na renda familiar da família da criança. Avaliamos 717 estudantes, 12,2% dos quais eram obesos. A variável latente ambiente obesogênico (deduzida das taxas de insegurança ambiental e do índice de estabelecimentos que comercializam majoritariamente alimentos ultraprocessados) constituiu o fator de risco para obesidade em crianças em famílias de baixa renda (OR = 2,37; IC95%: 1,06-5,19). Parques e espaços públicos para a prática de atividade física foram fatores de proteção contra a obesidade infantil apenas nos locais que registraram as menores taxas de insegurança ambiental (OR = 0,30; IC95%: 0,09-0,94). Com base em nossos achados, a condição social modifica o efeito do ambiente sobre a obesidade infantil e reforça a relevância de políticas e estratégias intersetoriais para prevenir a obesidade infantil.

## Resumen

Este artículo busca analizar los efectos aislados y combinados de medidas objetivas relacionadas con la seguridad del vecindario, la alimentación y los ambientes de actividad física sobre la obesidad de los niños en edad escolar. Este estudio transversal fue realizado con niños de 9 y 10 años de edad que estaban matriculados en la red municipal de enseñanza de una metrópoli brasileña. Las medidas objetivas ambientales incluyeron la inseguridad en los barrios (índices anuales de criminalidad y accidentes de tránsito), disponibilidad de espacios públicos para la práctica de actividad física y el índice de establecimientos que comercializan mayoritariamente alimentos ultraprocesados. Se tomó como unidad geográfica elegible un *buffer* euclidiano de 1.000 metros en torno a la casa de los niños. Nuestro análisis abarcó los modelos de Análisis de Componentes Principales y Estimación de Ecuaciones Generalizadas. Se realizaron análisis estratificados basados en la inseguridad del vecindario y en los ingresos de la familia del niño. Evaluamos a 717 estudiantes, de los cuales el 12,2% eran obesos. La variable latente ambiente obesogénico (deducida de las tasas de inseguridad ambiental y del índice de establecimientos que comercializan mayoritariamente alimentos ultraprocesados) constituyó el factor de riesgo de obesidad en niños de familias con bajos ingresos (OR = 2,37; IC95%: 1,06-5,19). Los parques y espacios públicos para la práctica de actividad física fueron factores de protección contra la obesidad infantil solo en los lugares que registraron las menores tasas de inseguridad ambiental (OR = 0,30; IC95%: 0,09-0,94). Sobre la base de nuestros hallazgos, la condición social modifica el efecto del ambiente sobre la obesidad infantil y refuerza la relevancia de las políticas y estrategias intersectoriales para prevenir la obesidad infantil.

## Introduction

Obesity prevalence has been increasing in a worrisome way among children in the last four decades. Such scenario features childhood obesity as one of the main public health concerns of present times ^1^. The *Brazilian Household Budget Survey*, 2008-2009 (POF 2008-2009) has pointed toward a significant increase in obesity rates in comparison to surveys performed in 1974-1975. It increased from 2.9% to 16.6%, and from 1.8% to 11.8%, among children aged 5-9 years, respectively ^2^. 

Socio-ecological models suggest that childhood obesity derives from a complex series of individual and environmental factors that relying on family, community, school, social, and political contexts ^3^ ^,^ ^4^.

Neighborhood safety around children’s homes (assessed based on criminality and on road traffic accident rates), as well as built environment factors such as public spaces for practicing physical activity (sports equipment in public locations, parks, trails, among others) and community food environment (availability of food outlets) have been associated with obesity ^5^ ^,^ ^6^ ^,^ ^7^. Environments are classified as obesogenic when they can increase the chances of weight gain by discouraging the practice of physical activity, facilitating adoption of a sedentary behavior, and influencing unhealthy eating habits ^8^.

Systematic reviews have shown inconsistent results regarding the influence of neighborhood and built environment on childhood obesity, but it is essential highlighting that most studies in this field were conducted in Northern hemisphere countries ^5^ ^,^ ^6^ ^,^ ^7^. Such inconsistent results can be attributed to methodological differences, as well as to contextual and cultural differences ^9^. Furthermore, it is essential to consider the potential impact of combined presence, or lack, of a whole series of contextual features linked to childhood obesity, rather than trying to identify the potential contribution of each separate variable or indicator since environmental factors are complex and interact with each other in different ways ^7^ ^,^ ^10^.

Moreover, in the statistical modeling of studies that assess the effect of the environment, it is relevant to consider social conditions, such as individuals’ socioeconomic features and the neighborhood safety, as potential modifiers to the effect of the environment. Individuals facing the worst socioeconomic conditions tend to be more sensitive to the effects of the environment they live than the ones who present a better socioeconomic condition and who have autonomous mobility and own transportation means ^7^ ^,^ ^11^. Environment safety level can also affect the effectivity of public equipment on childhood obesity since the fear of violence and road traffic accidents in large cities have been factors associated with low physical activity rates ^12^.

In this context, more evidence about this topic are necessary for further development of policies aimed at changing the environment and preventing childhood obesity ^13^ ^,^ ^14^. It is worth highlighting that there are few studies evaluating the combined effect of contextual features on childhood obesity ^7^ ^,^ ^15^ ^,^ ^16^. Therefore, this study aimed to analyze the isolated and combined effect of objective measures of neighborhood safety, physical activity, and food environments, as well as the role of social status as a modifier of these associations.

## Methods

### Sampling and study design

This is a cross-sectional study conducted with students enrolled in the fourth grade of elementary schools of a municipal education network (almost all participants were aged 9-10 years/94.5% of the sample) from a state capital in Southeastern Brazil (Belo Horizonte, Minas Gerais State). Data were collected from August 2014 to May 2015. Based on the last Demographic Census (2010) ^17^, 2,375,151 people live in Belo Horizonte, totaling 487 neighborhoods, and 7,167.00 inhabitants/km^2^ of demographic density, along with nine administrative regions, which are featured by socioeconomic contrasts ^18^.

Sample size was estimated based on the list provided by the Department of Education of Belo Horizonte, with 10,623 students enrolled in 424 classes of the 4th grade of the Elementary School, in 136 municipal public schools. The 50% ratio was considered for the sample (value that results in the largest sample size), for a given feature (multiple outcomes were taken into consideration), at 5% significance level (alpha or type I error), and 5% sampling error, based on criteria by Hulley et al. ^19^. Thus, the minimum sampling size estimated for this study comprised 371 participants. Then, a decision was made to broaden the selection by approximately 2.5 times the minimum sampling number estimated for the study; thus, it totaled approximately 928 participants. This strategy was based on previous experiences concerning participation rates available in the literature about research conducted with students, mainly the ones encompassing parents and guardians ^20^.

Sampling design was featured by two-stage cluster sampling (schools and classes) and proportional stratification per location. Therefore, 17 schools were randomly drawn - the raffle respected the size of the municipality’s nine administrative regions (stratified raffles were proportional to size, without reposition), so that the number of students enrolled in the fourth grade of these elementary schools reached 931 children, who were distributed into 34 classes - all these children were invited to participate in the research. 

Children who have missed school in the day of data collection were excluded from the research (n = 101), as well as students who have refused to participate in it (n = 2), the ones whose mothers/guardians did not allowed participation in the study (n = 36), or those who had impaired mental health - based on teachers’ reports - (n = 31). A total of 44 of the 761 assessed students were excluded from this study since they did not live in Belo Horizonte. Furthermore, data of students who belonged to census sectors lacking income information based on Brazilian Institute of Geography and Statistics’s (IBGE) *Demographic Census* were also excluded from the analysis (n = 10).

Accordingly, the final sample was composed of 707 students. Students excluded from the study did not present significant statistical differences from the ones who were in the sample regarding gender, age, and residence region in the municipality (p > 0.05) (data collected from school documents). Students who were excluded from the study since they did not live in Belo Horizonte or lived in census tracts without income information also did not present significant statistical differences in the classification of nutritional status (p > 0.05).

### Socioeconomic data 

Information about gender, date of birth (used to calculate age), school time (morning/afternoon shifts or day shifts), home address, and landline number were collected from school records.

For the collection of socioeconomic data, a questionnaire was applied to the parents or guardians by telephone call. Only questions regarding monthly family income and number of residents per household were considered in the questionnaire’s block of socioeconomic information. 

The ratio between all monthly incomes and the total number of households was calculated to estimate monthly family income per capita. Simple income per capita was considered for individuals assessed in 2014, and deflated income was obtained for the ones assessed in 2015. Deflated income was calculated by the ratio between per capita income and Deflation Index [ratio between minimum wage value in 2015 (BRL 788.00) and the minimum wage in 2014 (BRL 724.00)]. In total, 408 (56.9%) of the 717 children included in the study missed socioeconomic data due to the impossibility of contacting their parents/guardians due to not having a phone contact (n = 14) or wrong/unavailable phone number (n = 394). Three attempts were made to contact the parent or guardian by telephone call. The missing data of monthly incomes were estimated using the Multiple Imputation method. In this analysis, information from the Health Vulnerability Index (HVI) was considered; this is a synthetic indicator composed of socioeconomic and basic sanitation variables concerning the census sector where the child’s residence is located in. Thus, 20 complete databases were created with input results and they generated likely values for this variable. This analysis was conducted using Stata statistical program, version 12.0 (https://www.stata.com); commands such as “mi impute” - which creates inputs for predictive Bayesian distribution simulation - and “mi estimate” - which makes individual analyses of the complete database and consolidates the individual estimates into a single value - were used in the research.

### Childhood obesity 

The anthropometric assessment of the children was conducted at school. Students’ anthropometry included weight and height measurements. The collected data were used to calculate BMI [weight(kg)/height(meters)^2^]-per-age, based on the z-score, in WHO Anthro Plus software (version 3.2.2, https://who-anthro.software.informer.com/3.2/). Children with values higher than z score + 2 for this index were considered with obesity ^21^ ^,^ ^22^. This dichotomous variable was considered as the study dependent variable.

### Environmental variables

Mean monthly income per residence (contextual income); availability of public parks and sports practicing equipment; availability of food outlets; and annual criminality and road traffic accidents rates in the neighborhood were the environmental variables assessed. 

Data provided by the IBGE about the 2010 *Demographic Census* (https://www.ibge.gov.br/) were used to build the contextual income variable. Variable “total of nominal income of the head of the household” was divided by variable “number of people living in the household” to calculate mean monthly income per residence in the census sector. The mean contextual income was calculated using the mean monthly income per residence of the census sector whose centroid was within the 1,000m buffer around the residence of each child. 

Information about public parks and spaces for physical activity practicing, and of their respective addresses, which were provided by Belo Horizonte’s City Hall (https://prefeitura.pbh.gov.br), were also collected. Thus, data about municipal parks, outdoor gyms (in public spaces such as squares and gardens across the city, where equipment are used by people at different age groups), and sports practicing infrastructure (soccer fields, playgrounds, skate pools, tennis courts, trails, among others) were considered. The density/population of these public parks and spaces for physical activity practicing (number of pieces of equipment for each 10,000 inhabitants) within each 1,000m buffer around the child’s residence was calculated. 

A database for the availability of establishments selling food was built by considering address and the Brazilian National Classification of Economic Activities (CNAE), of 11 types of establishments in Belo Horizonte city that were registered in 2015, namely: hypermarkets, supermarkets, grocery stores, bakeries, dairy products and delicatessen shops, candy shops, butchers, fish shops, restaurants, and snack bars. This set of information was collected from two sources: Superintendence of Finance and Information of the Minas Gerais State (n = 17,103 establishments) and the Municipal Inspection Department (n = 10,192 establishments). Establishments that did not fit these two databases (n = 12,517) were categorized using the Google Street View tool, which is available in the Google Maps app - it allows for the panoramic view of the streets (https://www.google.com.br/maps). Detailed information regarding the process of database construction have been described in an article by Rocha et al. ^23^. The final database consisted of matching establishments between the two secondary databases, along with those found in the Google Street View. Moreover, this database was supplemented with information about Belo Horizonte City Hall’s establishments that trade fruits and vegetables, namely: grocery suppliers, *Direto da Roça* (a Brazilian organic food program) and organic products free fairs (https://prefeitura.pbh.gov.br). 

These data allowed building the index of establishments that predominantly sell ultra-processed food (%) based on the following calculation: (establishments that predominantly sell ultra-processed food/total number of food shops within each 1,000m buffer around the child’s residence) x 100. Snack bars and candy shops were classified as establishments that predominantly sell ultra-processed food, following another study called *Mapping Food Deserts in Brazil* ^24^.

Geographic coordinates (latitude and longitude) of the records of road traffic accidents and violence (including rape, extortion during kidnap, homicides, robbery, kidnapping, and torture) in Belo Horizonte, in 2014 and 2015, were provided by Integrated Center of Social Defense Operations. These data were possible by calculating the variables’ annual rates per 10,000 inhabitants in 2014 and 2015, and by considering the geographic unit “1,000m buffer”, along with the total population (variable: households in private and collective houses) provided by the 2010 IBGE’s *Demographic Census* - its centroid was located within the 1,000m buffer. 

All the aforementioned contextual variables were categorized into distribution quartiles for the analysis. The contextual income variable was used as adjustment in the statistical models. The availability of public parks and sports practicing equipment, proportional availability of establishments that predominantly sell ultra-processed food, and annual criminality and road traffic accidents rates in the neighborhood were used as explanatory variables in the models.

### Data analysis 

#### Geo-processing environment variables

The spatial location (latitude and longitude) of the children’s home addresses and of the built environment was obtained using the free R programming language, version 3.4.4 (http://www.r-project.org), which geo-codifies addresses by using the location service available at Google Maps. Subsequently, all the collected information was geo-referenced and treated in QGIS software, version 2.10.1 (https://qgis.org/en/site). 

Round buffers were drawn around the children’s residences. These buffers were based on a 1,000m radius, which corresponds to approximately 12 to 15-minute walk, and to 1 to 2-minute car ride ^25^; points are approximately centered on geographic points that represent each residence. These buffers were considered the social and physical surroundings of participants’ homes (eligible geographic unit). These data were added to individual data of participants in the sample to create a single database. 

#### Statistical analysis

**Figure 1:**
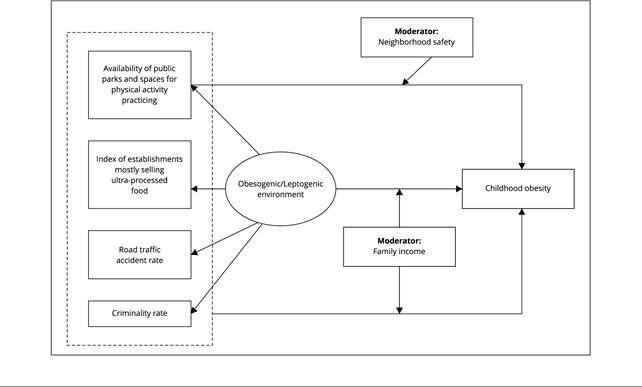
Hypothesized theoretical model to set the association between environmental objective measures and child obesity, and the role of potential association changers.

Note: adjustment variables are not represented in this model.

Figure 1 depicts the theoretical model to be herein tested. According to this model, environment variables can influence childhood obesity in either isolate or synergic way, given the combined effect of these variables. Such an effect forms the latent variable of the environment that, in its turn, can be obesogenic or leptogenic to child nutritional status, depending on the features of the contextual variables. Latent variables are constructs that cannot be directly observable and that can be indirectly inferred based on indicators (observed variables) ^26^. This model suggests that the effect of neighborhood variables on childhood obesity depend on children’s family income. Moreover, this model suggests that the effect of parks and public sports practicing spaces also depend on safety level (these factors were assessed by summing the rates of road traffic accidents to criminality rates in the neighborhood).

Regarding data analysis, descriptive analysis was conducted based on relative frequency, mean, and interquartile range distribution (percentile 25 and 75) calculations. Chi-square test and Mann-Whitney were used to compare ratios and mean, respectively. 

The principal components analysis was carried out to explore the combined effect of contextual features by forming the latent variable using the observed variables of the environment; it was performed by finding Kaiser-Meyer-Olkin (KMO) index as adequacy measurement of the factorial analysis - values ranging from 0.5 to 1.0 recorded for this index were considered acceptable ^26^. Factorial load higher than 0.4 was considered for the standardized estimate analyses applied to the latent variables ^27^.

Analysis of the results (Supplementary Material: https://cadernos.ensp.fiocruz.br/static//arquivo/suppl-e00104822_9472.pdf) have suggested the formation of only one principal component, which has contributed to 56.7% variance in total information. The KMO index was 0.660; this number points to satisfactory value. The factorial loads of all variables were satisfactory (> 0.40), except for “public parks and spaces for physical activity practicing”; which was 0.027. 

After excluding the explained variance of the principal component, it increased to 75.6% and the KMO index rose to 0.662. Finally, all variables in this final stage (index of establishments that predominantly sell ultra-processed food, road traffic accident rate, and criminality rate in the neighborhood), which have composed the latent variable “obesogenic environment” (Cronbach’s alpha = 0.766), have presented satisfactory factorial load and positive values. This variable was categorized based on the distribution of percentile 50. Thus, scores higher than or equal to the mean represent the most obesogenic environments.

Generalized Estimating Equations (GEE) using logistic regression model, which generates efficient estimates for regression parameters, based on correlated data ^28^, were used as model since most assessed children (91.5%) shared the same neighborhood (there was one child per neighborhood in 61 neighborhoods, and, on average, 4.8 children per neighborhood in 159 neighborhoods). The odds ratios (OR) and their respective 95% confidence intervals (95% CI) were estimated.

Unadjusted and adjusted associations were performed between the observed contextual variables and the latent construct “obesogenic environment”, along with childhood obesity. Stratified analyses were performed based on the changing potential of the effect. Models were run to assess the association between contextual variables and childhood obesity, stratified by monthly family income, and the association between “parks and public sports practicing spaces” and obesity, stratified by environment unsafety. The adjusted variables included in the study were gender, school time, monthly family income per capita, and contextual income. 

All analyses were conducted in Stata program, version 12.0. Significance level of 5% was considered for the analyses.

### Ethical aspects

Research protocol was approved by the Research Ethics Committee oh the Federal University of Minas Gerais (CAAE 00734412.0.0000.5149). All children’s parents and/or guardians, as well as the assessed students, received and signed an informed consent form to respect their dignity and integrity. 

## Results 

In total, we assessed 707 students, with mean age of 9.7 years old (9.4:10.0), 51.3% of them were girls, and 12.2% were children with obesity. Table 1 describes the individual and the environmental variables. We found no association between childhood obesity and gender, age, school time, and contextual income (p > 0.05). Children with obesity recorded the highest mean family income per capita value (BRL 440.60 vs. BRL 379.10, p = 0.002) (Table 2). Students belonging to the highest-income families lived in environments with higher records of criminality and road traffic accident rates, higher mean contextual income values, and higher indices of establishments that predominantly sell ultra-processed food (p < 0.05) (data not shown).

**Table 1:** Characteristics of the assessed sample. Belo Horizonte, Minas Gerais State, Brazil, 2014-2015 (n = 707).

Characteristics	Total	Body mass index per age		p-value *
		Without obesity	With obesity	
Children				
% girls	51.3	52.2	41.1	0.057
% day shift school	45.0	45.3	42.5	0.611
Age (years)	9.7 (9.4-10.0)	9.7 (9.4-10.0)	9.8 (9.5-10.0)	0.256
Monthly family income per capita (BRL)	386.0 (291.7-464.9)	379.1 (290.4-460.1)	440.6 (334.9-496.4)	0.002
Environmental **				
Mean contextual income (BRL)	1,139.9 (919.7-2,146.3)	1,120.6 (914.3-2,119.0)	1,228.5 (953.6-2,280.1)	0.394
Criminality (annual rate per 10,000 inhabitants)	100.9 (64.9-171.1)	100.6 (64.2-166.0)	122.5 (72.5-200.6)	0.099
Road traffic accidents (annual rate per 10,000 inhabitants)	122.1 (72.2-204.1)	119.8 (72.2-200.5)	135.2 (74.4-236.6)	0.155
Environment unsafety (annual rate per 10,000 habitants) ***	225.3 (153.2-377.5)	221.1 (152.4-371.1)	277.1 (160.4-436.8)	0.102
Public parks and spaces for physical activity (counting per 10,000 inhabitants)	2.0 (1.5-2.7)	2.0 (1.5-2.7)	2.0 (1.4-2.8)	0.831
Index of establishments that predominantly sell ultra-processed food (%) ^#^	30.0 (23.7-34.0)	30.0 (23.6-33.9)	30.2 (26.1-34.5)	0.208

Note: values presented as relative frequency (%) and median (p25-p75) for categorical and quantitative variables, respectively.

* Mann-Whitney test and chi-square test to compare medians and ratios, respectively;

** Environmental factors based on the 1,000m buffer around children’s homes as geographic unit;

*** Sum of annual rates (per 1,000 inhabitants) of the number of crimes and road traffic accidents;

^#^ (Establishments that predominantly sell ultra-processed food/total number of food shops) x 100.

**Table 2:** Contextual factors associated with childhood obesity: analysis without stratification based on monthly family income per capita. Belo Horizonte, Minas Gerais State, Brazil, 2014-2015 (n = 707).

Environmental characteristics	Total sample		**Monthly family income *per capita***			
			< median		≥ median	
	Crude OR (95%CI)	Adjusted OR * (95%CI)	Crude OR (95%CI)	Adjusted OR ** (95%CI)	Crude OR (95%CI)	Adjusted OR ** (95%CI)
Crimes/10,000 inhabitants						
Quartile 1	Reference	Reference	Reference	Reference	Reference	Reference
Quartile 2	1.01 (0.52-1.99)	0.86 (0.41-1.80)	1.08 (0.40-2.89)	0.82 (0.28-2.39)	0.82 (0.33-2.00)	0.82 (0.29-2.25)
Quartile 3	1.11 (0.57-2.17)	0.98 (0.48-2.01)	1.71 (0.70-4.18)	1.29 (0.48-3.49)	0.58 (0.22-1.33)	0.58 (0.21-1.59)
Quartile 4	1.40 (0.73-2.66)	1.04 (0.46-2.34)	1.10 (0.28-4.28)	0.86 (0.19-3.82)	0.97 (0.44-2.15)	0.93 (0.33-2.59)
Road traffic accidents/10,000 inhabitants						
Quartile 1	Reference	Reference	Reference	Reference	Reference	Reference
Quartile 2	0.92 (0.47-1.80)	0.78 (0.38-1.57)	1.15 (0.45-2.91)	1.00 (0.38-2.62)	0.50 (0.19-1.29)	0.46 (0.17-1.21)
Quartile 3	0.92 (0.46-1.81)	0.74 (0.33-1.67)	0.91 (0.32-2.60)	0.95 (0.29-3.04)	0.56 (0.22-1.41)	0.53 (0.18-1.49)
Quartile 4	1.48 (0.80-2.75)	1.17 (0.50-2.76)	1.46 (0.51-4.72)	1.56 (0.40-6.02)	0.90 (0.39-2.09)	0.6 (0.30-2.43)
Environment unsafety/10,000 inhabitants ***						
Quartile 1	Reference	Reference	Reference	Reference	Reference	Reference
Quartile 2	0.76 (0.39-1.49)	0.55 (0.25-1.19)	1.20 (0.45-3.20)	0.86 (0.27-2.68)	0.52 (0.20-1.33)	0.34 (0.11-1.09)
Quartile 3	0.97 (0.51-1.86)	0.76 (0.35-1.64)	1.73 (0.70-4.24)	1.48 (0.53-4.13)	0.59 (0.23-1.50)	0.42 (0.13-1.34)
Quartile 4	1.40 (0.75-2.58)	0.99 (0.41-2.38)	1.36 (0.40-4.62)	1.41 (0.30-6.59)	0.93 (0.42-2.04)	0.61 (0.18-2.02)
Public parks and spaces for physical activity/10,000 inhabitants						
Quartile 1	Reference	Reference	Reference	Reference	Reference	Reference
Quartile 2	0.88 (0.47-1.54)	0.78 (0.41-1.49)	0.70 (0.27-1.81)	0.50 (0.18-1.38)	1.08 (0.46-2.50)	1.05 (0.44-2.47)
Quartile 3	0.71 (0.36-1.40)	0.59 (0.29-1.20)	0.45 (0.15-1.33)	0.39 (0.12-1.22)	1.06 (0.46-2.47)	0.93 (0.38-2.23)
Quartile 4	0.82 (0.43-1.56)	0.70 (0.35-1.37)	0.59 (0.22-1.57)	0.46 (0.16-1.34)	1.11 (0.48-2.55)	0.98 (0.41-2.35)
Index of establishments that predominantly sell ultra-processed food (%) ^#^						
Quartile 1	Reference	Reference	Reference	Reference	Reference	Reference
Quartile 2	1.58 (0.80-3.12)	1.35 (0.67-2.73)	1.83 (0.63-5.25)	1.88 (0.66-5.37)	1.15 (0.47-2.82)	1.00 (0.39-2.57)
Quartile 3	1.25 (0.62-2.52)	1.09 (0.52-2.27)	1.53 (0.52-4.54)	1.67 (0.55-5.03)	0.89 (0.35-2.26)	0.78 (0.30-2.04)
Quartile 4	1.67 (0.85-3.28)	1.33 (0.63-2.82)	2.28 (0.77-6.79)	2.21 (0.68-3.10)	1.07 (0.44-2.56)	0.98 (0.38-2.53)
Obesogenic environment ^##^						
< median score	Reference	Reference	Reference	Reference	Reference	Reference
≥ median score	1.56 (0.97-2.48)	1.48 (0.85-2.59)	2.09 (1.01-4.32) ^###^	2.37 (1.06-5.19) ^###^	1.12 (0.61-2.06)	1.04 (0.57-1.89)

95%CI: 95% confidence interval; OR: odds ratio.

Note: dependent variables of the models: child obesity.

* Adjusted by gender, time staying in school, monthly family income per capita, and mean contextual income;

** Adjusted by gender, time staying in school, and mean contextual income;

*** Sum of annual rates (per 1,000 inhabitants) of the number of crimes and road traffic accidents;

^#^ (Establishments that predominantly sell ultra-processed food/total number of food shops) x 100;

^##^ Latent variables deduced by the variables: ratio of establishments mostly selling ultra-processed food, criminality rate, rate of road traffic accidents. Scores lower than the median represent lesser obesogenic environments, and scores higher than, or equal to, the median represent the most obesogenic environments;

^###^ p < 0.05.

We found no significant association of any contextual variable with childhood obesity when they were separately tested after adjustment based on gender, age, school time, family income and mean contextual income (p < 0.05) (Table 2). Considering analyses stratified by monthly family income, it is possible to observe that, among children from families with lower income, those who live in a more obesogenic environment - a latent variable deduced by environment unsafety and by the index of establishments that predominantly sell ultra-processed food - had 2.37 times more chances of obesity, after the adjustment, based on individual and contextual variables (95%CI: 1.06-5.19) (Table 2). Among children from families with higher incomes, this association was not significant (p > 0.05).

The association between parks and public sports practicing spaces and childhood obesity based on environment unsafety rate is described in Table 3. Our results suggest that parks and spaces for physical activity were protective factors against child obesity only in the safest neighborhoods. Children living in the safest locations and in the largest quartile of available public parks and spaces for physical activity have recorded lower chances of obesity (OR = 0.30; 95%CI: 0.09-0.94). Association between these public spaces and obesity was not significant among children living in the most unsafe locations (p > 0.05).

**Table 3:** Logistic regression analysis between public parks and spaces for physical activity, based on the environment unsafety rates. Belo Horizonte, Minas Gerais State, Brazil, 2014-2015 (n = 707).

Explanatory variable	Environment unsafety rate (< median) *		Environment unsafety rate (≥ median) *	
	Crude OR (95%CI)	Adjusted OR (95%CI) **	Crude OR (95%CI)	Adjusted OR (95%CI) **
Public parks and spaces for physical activity /10,000 inhabitants				
Quartile 1	Reference	Reference	Reference	Reference
Quartile 2	0.69 (0.27-1.77)	0.62 (0.23-1.68)	1.04 (0.44-2.43)	0.94 (0.40-2.20)
Quartile 3	0.67 (0.26-1.73)	0.54 (0.19-1.54)	0.70 (0.26-1.87)	0.71 (0.28-1.80)
Quartile 4	0.42 (0.14-1.19)	0.30 (0.09-0.94) ***	1.37 (0.58-3.20)	1.39 (0.57-3.35)

95%CI: 95% confidence interval; OR: odds ratio.

Note: dependent variable of the models: child obesity.

* Sum of annual rates (per 1,000 inhabitants) of the number of crimes and road traffic accidents;

** Adjusted by gender, time staying in school, monthly family income per capita, and mean contextual income;

*** p < 0.05.

## Discussion

Our outcomes have shown the relevance of analyzing the effect of grouping contextual variables, including the changing potential of the association between environment and childhood obesity. Variables “criminality”, “road traffic accidents”, “availability of public parks and spaces for physical activity practicing”, and “index of establishments that predominantly sell ultra-processed food” did not present association with childhood obesity when they were separately analyzed. However, we noted significant associations with childhood obesity when combinations between these variables and the stratified analyses were tested, based on family income and environment unsafety. 

Obesity is a multi-factorial condition; it is the outcome of the complex interaction of different determinants ^29^. In fact, based on our outcomes, the combined effect of the studied environmental factors explained childhood obesity through the latent variable “obesogenic environment” rather than through the isolated effect of each of these factors.

Research that had been addressing the combined effect of environmental factors have presented more consistent results with childhood and adolescent obesity ^7^ ^,^ ^15^ ^,^ ^16^. A study conducted in San Diego (United States) recorded higher overweight and obesity rates in children living in unfavorable environment either for healthy eating or for exercising ^30^. A cluster analysis study conducted with Portuguese students evinced that living in neighborhoods with new buildings and more green areas was a protective factor against obesity ^16^.

The higher proportion of establishments that predominantly sell ultra-processed foods was one of the factors that characterized the latent variable “obesogenic environment”, which, in turn, was associated with a greater chance of childhood obesity. An ecological study conducted in the city of Belo Horizonte showed that throughout 10 years (2008-2018), changes in the neighborhood food environment were unfavorable for adequate access to healthy foods, especially in lower-income neighborhoods ^31^. Another study showed that food deserts and swamps were widely found, often in the same areas in Belo Horizonte, and also found to be more frequent in the lowest-income areas ^32^. These results indicate the importance of public policies that seek to promote healthy food supply to prioritize the most vulnerable areas, with poor access and availability of food ^31^.

Our results point to the potential of availability of public parks and spaces for physical activity as protective factors against childhood obesity in large Brazilian cities. Furthermore, this finding indicate that the use of these locations were more favorable within the safest neighborhood buffers. A similar result was found for North American children, in which the presence of urban recreational trails in neighborhoods was a protective factor against obesity only in low-criminality regions ^12^. Another cross-sectional study based on a nationally representative sample of students enrolled in the 5th grade of elementary school in the United States has shown that children living in neighborhoods considered unsafe were less prone to use leisure facilities and also presented lower exercising rates than the ones living in neighborhoods considered safe ^33^. These findings have highlighted the importance of investing in effective strategies and public policies focused on reducing criminality and road traffic accidents, rather than only investing on leisure and public physical activity infrastructures.

Road traffic accident and criminality rates are quite high in Brazilian cities, mainly in large cities. Brazil is one of the most violent countries in the world; it records more than 28 homicides for each 100,000 inhabitants ^34^. Actions taken to reduce traffic accidents and criminality must be substantiated by preventive measures applied to their roots, including society, family, and individuals, and by urbanization and security policies aimed at creating safer environments ^34^.

According to our results, the latent variable “obesogenic environment” was a risk factor for childhood obesity only among children belonging to lower-income families, which corroborates with findings in other national and international studies. A study conducted in the United States has shown that children living in low-income cities presented more consistent association between the environment construct and obesity ^11^. Similarly, a study found that living closer to parks/playgrounds was associated with lower BMI only in students from low-income families from Florianópolis city, Santa Catarina State, Brazil ^35^.

Assumingly, healthy behaviors and, consequently, the body weight of children in lower-socioeconomic level are factors more dependent on local environment determinants than their peers in higher socioeconomic levels, since the former mainly rely on the infrastructure in the neighborhood ^7^ ^,^ ^9^. Therefore, policies focused on reversing obesogenic environments can be a protective factor against childhood obesity in poor families. 

The aforementioned findings support the recommendation that obesity prevention in children, as well as in adults, demands inter-sectoral policies aimed at increasing neighborhoods safety, providing easier access to equipment that allow physical activity, and encouraging establishments to sell healthy food ^8^. Therefore, the support for individuals to change obesogenic behaviors remain important, but public policies focused on structural changes should also be adopted ^8^.

Furthermore, our outcomes highlight that social condition can change the environment effect and the scenario of socioeconomic inequalities associated with the highest chance of developing child obesity when analyzing the stratifications based on changer potentials. Therefore, this scenario must considered when planning intervention strategies and public policies. 

This study presents limitations, such as the cross-sectional design-, which does not allow cause/effect conclusions, and sample homogeneity regarding socioeconomic aspects since only public school students were assessed. Lack of variability can also explain some non-significant associations. However, our study is relevant due to the sample representativeness of the nine administrative areas of Belo Horizonte, which are featured by socioeconomic contrasts ^18^.

Another limitation of our study lies on the non-inclusion of some food shops that were not registered in any of the two secondary databases (true negative) - this problem could be solved by observation in loco. However, this method cannot be applied for the entire Belo Horizonte, given its large extension; such observations are recommended for small regions only ^36^. Assumingly, these non-registered establishments are more common in more economically vulnerable locations ^37^. This study also assessed the food environment around residences, but it did not assess other locations where parents/guardians can likely go to buy food (locations nearby parents’ work places, among others). The collection and combination of information about where and when parents go grocery shopping, as well as the network buffers approach, instead of the Euclidean one, should be explored in further research.

Another limitation was that we did not consider the school environment, which could be relevant since children spend much of their time within that environment. Some studies conducted with Brazilian children and adolescents samples showed an association between school environment variables and outcomes such as obesity ^38^ and cardiometabolic risk ^39^.

We highlight that criminality and road traffic accident data must be carefully interpreted since they are open to a whole series of validity and reliability limitations. These data often depict the social process of notification rather than providing an accurate representation of the entire situation in a specific area. In addition, although individuals likely perceive neighborhoods as the area including their homes and surrounding spaces, the used geographic unity (1,000m buffer) may not reflect participants’ perception about the neighborhood in the assessed geographic area.

Despite the presented limitations, it is worth highlighting the potential of the study, which includes using objective environmental measures and methodology to develop a food environment database, increasing information reliability. Furthermore, our findings show the combined effect of environment factors and how family income and neighborhood safety change associations between contextual features, public parks and spaces for physical activity, and childhood obesity.

## Conclusion 

Our findings suggest that parks and spaces for physical activity were protective factors against childhood obesity only in the safest neighborhoods. The interaction between environmental factors was also important to explain obesity. The obesogenic environment - a latent variable deduced by environment unsafety and the index of establishments that predominantly sell ultra-processed food - was a risk factor for obesity, but this association only took place among children belonging to families with lower socioeconomic levels. Thus, these results point to the economic and social inequalities that are associated with greater risk of developing childhood obesity.

Therefore, policies and strategies developed by several governmental sectors should aim at changing these environmental obesogenic features that are relevant to prevent childhood obesity, mainly focusing students who present worst socioeconomic conditions. Furthermore, based on our results, governmental actions focused on reducing criminality and traffic accidents can be essential to potentiate the use of public parks and spaces for physical activity.
